# Generation of Mature DENVs via Genetic Modification and Directed Evolution

**DOI:** 10.1128/mbio.00386-22

**Published:** 2022-04-28

**Authors:** Longping V. Tse, Rita M. Meganck, Stephanie Dong, Lily E. Adams, Laura J. White, Michael L. Mallory, Ramesh Jadi, Aravinda M. de Silva, Ralph S. Baric

**Affiliations:** a Department of Epidemiology, The University of North Carolina at Chapel Hill, Chapel Hill, North Carolina, USA; b Department of Microbiology and Immunology, The University of North Carolina at Chapel Hill, Chapel Hill, North Carolina, USA; Georgia State University; St. Jude Children’s Research Hospital

**Keywords:** antigenicity, dengue virus, directed evolution, maturation, viral engineering

## Abstract

Maturation of dengue viruses (DENVs) alters the structure, immunity, and infectivity of the virion and highly mature particles represent the dominant form *in vivo*. The production of highly mature virions principally relies on the structure and function of the viral premature membrane protein (prM) and its cleavage by the host protease furin. We redeveloped a reliable clonal cell line (VF1) which produces single-round mature DENVs without the need for DENV reverse genetics. More importantly, using protein engineering and directed evolution of the prM cleavage site, we engineered genetically stable mature DENVs in all serotypes independent of cell or host, usually with minimal impact on viral yield. Using these complementary strategies to regulate maturation, we demonstrate that the resulting mature DENVs are antigenically distinct from their isogenic partially mature forms. Given the clinical importance of mature DENVs in immunity, our study provides reliable strategies and reagents for the production of stable, high-titer mature DENVs for DENV antibody neutralization and vaccination immunity studies. Biologically, our data from directed evolution across host species reveals distinct maturation-dependent selective pressures between mammalian and insect cells, verifying the substrate preference between mammalian and insect furin, while hinting at an evolutionary equilibrium of DENV prM cleavage site between its host and vector in nature.

## INTRODUCTION

Mosquito-borne dengue virus (DENV) is a major global public health threat causing ~400 million new cases of dengue infection annually (1, 2). Although the majority of cases occur in tropical and subtropical areas where the mosquito vectors are most concentrated, global warming, travel, and globalization have contributed to the worldwide spread and intermixing of the four DENV serotypes (3). Indeed, DENV infection has increased 30-fold between 1960 and 2010, with an upsurge of cases in the United States and Europe (4, 5). A hallmark of DENV pathogenesis is the possibility for antibody-dependent enhancement, which can progress to life-threatening dengue hemorrhagic fever/dengue shock syndrome upon secondary infection with a different serotype. Thus far, no approved antiviral treatments are available to treat DENV disease and the only approved vaccine, Dengvaxia, is no longer recommended for use in naive populations (6, 7).

The DENV virion structural proteins consist of capsid, envelope (E), and premembrane (prM) glycoproteins which undergo major conformational changes via the process of maturation. The infectious, mature DENV composed of 90 E homodimers lying flat in a “herringbone” structure and organized into a 50-nm icosahedral (T = pseudo 3) symmetry (8). In contrast, the noninfectious (9), immature DENV adopts a completely distinct structure as a 60-nm “spikey” sphere with 60 3-fold spikes (10, 11). A third form of DENV is represented by the partially mature/immature particles that adopt both mature and immature structure in different regions which generate limitless different topologies in the viral population (12). Because of the different topologies between mature and immature DENV, they are predicted to present different antigenic structures and antibody epitopes (13, 14). Although the biological functions of these interchanging maturation forms remain largely unknown, the maturation process is thought to provide key evolutionary advantages in virus infection, immunity, and antigenic variation (15, 16). While the maturation status of common laboratory DENV strains varies, a previous study showed that clinical isolates are typically more mature, arguing for the clinical importance of mature DENVs in human (17).

Proteolytic cleavage of viral membrane fusion proteins is a common strategy for temporal or spatial control of virus infection, ultimately affecting tropism and transmission (18, 19). In DENV, maturation is controlled by furin cleavage of the prM. Furin is a ubiquitously expressed host serine protease in the *trans*-Golgi network (TGN) (11, 20–22). Although also expressed in insects, vitellogenin (the insect homolog) has only 39% sequence identity compared to mammalian furin and is known to have a different substrate preference (23). Cleavage of prM releases the pr portion from the virion and triggers a rotation and collapse of E protein to form the mature virion (10, 24, 25). In addition to conformational change in proteins, lipids may also play a role in stabilizing the mature DENV structure (26, 27). Given the importance of furin in DENV maturation, mature and immature DENV can be produced experimentally using Vero or 293 cells overexpressing furin (28) and LOVO cells (lacking furin expression), respectively. Although useful to study antigenic properties between the mature and immature DENVs, these single-round maturation phenotypes are completely dependent on the production cell lines, which limits utility for *in vivo* studies. Alternatively, genetic alteration of DENV furin cleavage site optimality may intrinsically control the virus properties to be more mature or immature; however, the viral fitness of these genetically modified viruses is not guaranteed and, indeed, may be compromised.

In this study, we have redeveloped a clonal furin expressing cell line, VeroFurin-clone-1 (VF1) as a reliable tool for generation of phenotypically mature DENV of all serotypes. In addition, we also generated mature variants of DENV1, -2, and -4 with identical viral fitness by iterative genetic modification. We also generated a mature variant of DENV3, which shows identical fitness in insect cells but is attenuated in mammalian cells. To avoid multiple rounds of genetic modifications and potential fitness issues, as a proof of concept, we performed a single round of saturation mutagenesis at the DENV2 furin cleavage site and passaged the viral library on both mammalian and insect cells. Using this strategy, we successfully generated two highly mature DENV2 variants, DV2-C1 and DV2-V1, without compromising viral fitness. Interestingly, variants evolved from mammalian and insect cell lines show different levels of cleavage efficiency by mammalian furin. Our results support substrate preference between mammalian and insect furin and hint at an evolutionary role of DENV maturation in vertebrate and invertebrate cells. Overall, the present study provides critical reagents for the DENV field and further insight into DENV maturation and evolution for future investigations.

## RESULTS

### DENV maturity is serotype and host dependent.

**FIG 1:**
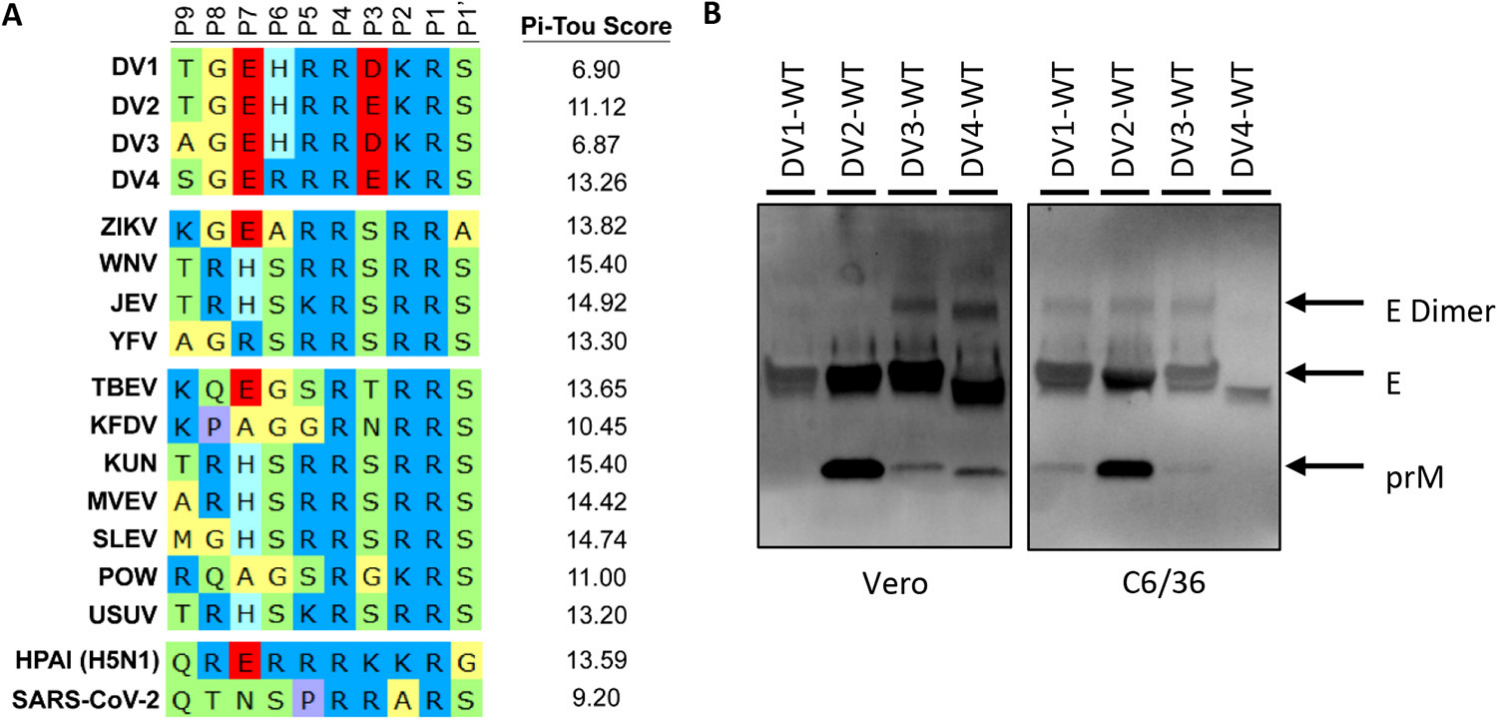
Furin cleavage site alignment and DENV maturation. (A) Amino acid sequence alignment of viral furin cleavage sites from position 9 (P9) to position 1 prime (P1′). PiTou scores are the prediction of logarithmic-odd probabilities of all the different viral furin cleavage sites (higher value = better substrate for furin). Viruses: DENV1, -2, -3, and -4; Zika virus (ZIKV); West Nile virus (WNV); Japanese encephalitis virus (JEV); yellow fever virus (YFV); Tick-borne encephalitis virus (TBEV); Kyasanur Forest disease virus (KFDV); Kunjin virus (KUN); Murray Valley encephalitis virus (MVEV); St. Louis encephalitis virus (SLEV); Powassan virus (POW); Usutu virus (USUV); highly pathogenic avian influenza virus (HPAI); SARS-Coronavirus-2. (B) Representative Western blot image of DV1-WT, DV2-WT, DV3-WT, and DV4-WT viral supernatants grown in either Vero or C6/36 blotted with anti-Env and anti-prM antibodies.

DENV maturation regulates virion infectivity and antigenicity and directly impacts antibody neutralization and potential vaccine efficacy. Because furin cleavage of the prM protein initiates the DENV maturation process, we hypothesized that furin cleavage efficiency is a major driver of DENV maturation. We compared the DENV1 to DENV4 prM cleavage site with other vector-borne flaviviruses. Sequence analysis suggested that all the DENV serotypes encoded a suboptimal furin cleavage site, (P4) R-X-K/R-R (P1), with negative modulators, as indicated by an acidic residue at the P3 position (Fig. 1A). To analyze the functionality of the prM furin cleavage site in a more quantitative manner, we used the computational program PiTou (http://www.nuolan.net/reference.html), which calculates the logarithmic-odd probabilities of different viral furin cleavage sites by mammalian furin (29). These analyses provided a reference that the DENV serotypes encode a less optimal furin sites (scores from 6.90 to 13.26) compared to other flaviviruses (scores from 13.30 to 15.40) (Fig. 1A). We focused our studies on four prototypical wild-type (WT) DENV viruses, including WestPac (DV1-WT), S16803 (DV2-WT), 3001 (DV3-WT), and Sri Lanka 92 (DV4-WT) isolates (Table 1). Using Western blotting as a readout, we determined the relative maturity of each serotype by calculating the ratio of prM to E. The relative maturity was clearly different between serotypes. In particular, serotypes encoding a glutamic acid (E) at the P3 position (prM residue 89) are associated with more immature virion production in Vero cells, with DV2-WT virions containing the highest level of uncleaved prM, followed by DV4-WT, DV3-WT, and DV1-WT, which has nearly undetectable levels of prM and hence is more mature (Fig. 1B). DENV maturation also depends on the producer cells; C6/36 grown DENVs show a different maturation profile, wherein DV4-WT is the most mature (Fig. 1B). PiTou predictions do not perfectly translate to the actual maturation status of DENV, indicating that prM cleavage is dependent on both local primary sequence and other distal and structural influences. As such, PiTou scores serve as a reference but not as a predictive parameter for the complexities of DENV maturation.

**TABLE 1:** DENV prototypes used in this study

Serotype	Strain	Genotype
DV1	WestPac 74 (WHO)	IV
DV2	S16803 (WHO)	Asian I
DV3	3001	III
DV4	Sri Lanka 92	IIb

### Clonal Vero-furin (VF1) cells generate high yield, phenotypic mature DENV.

**FIG 2:**
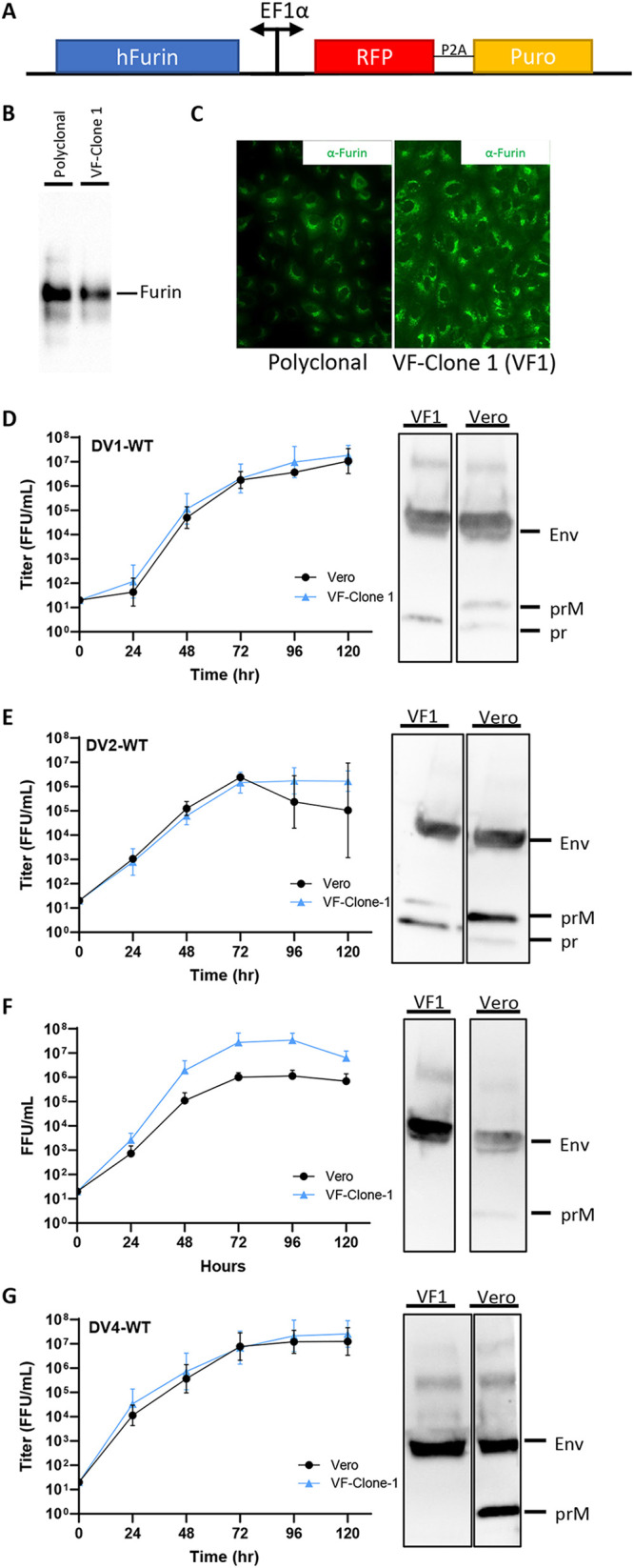
Growth kinetics and maturation status of DENVs on Vero and VF1 cells. (A) Schematic of the sleeping beauty-based transposon cassette for ectopic expression of human furin (hFurin). A bidirectional EF1a promoter was used to drive the expression of hFurin and red fluorescent protein (RFP) with a puromycin resistance gene (Puro) linked by a 2A self-cleaving peptide (P2A). (B and C) Western blot (B) and immunofluorescence (C) images of polyclonal and clonal VF1 using anti-furin antibodies. (D to G) Growth kinetics and degree of maturation of DENV1-WT (D), DENV2-WT (E), DENV3-WT (F), and DENV4-WT (G) in unmodified Vero cells (black circles) and VF1 cells (blue triangles). Cells were infected with DENV at an MOI of 0.01 to 0.05 for 120 h. Supernatants were harvested every 24 h for 120 h, and the 120-h supernatants were analyzed by Western blotting for DENV maturation using anti-Env and anti-prM antibodies. All assays were performed with at least two biological repeats with two technical replicates. Growth kinetics of DENV variants were compared to their corresponding wild type using two-way ANOVA multiple comparisons.

As reported previously (28), fully mature DENV can be generated in Vero cells that overexpress furin. Using the sleeping beauty transposon system (30), we generated a polyclonal cell line and subsequently a clonal line (VF1) with high levels of furin expression (Fig. 2A). Immunofluorescent staining and Western blot analysis revealed variable levels of furin expression in the *trans*-Golgi network of the polyclonal line but a uniform expression of the VF1 (Fig. 2B and C). The growth kinetics of all four DENV serotypes were tested on VF1 line and compared to unmodified Vero cells (Fig. 2D to G). DV1-WT, DV2-WT, and DV4-WT showed similar growth kinetics in all cell lines tested, while VF1 supported better DV3-WT growth (Fig. 2D to G). The redeveloped VF1 supports the production of fully mature DENV virions across all four serotypes (Fig. 2D to G).

### Genetic enhancement of DENV prM cleavage sites generates mature DENV1 and DENV2.

**FIG 3:**
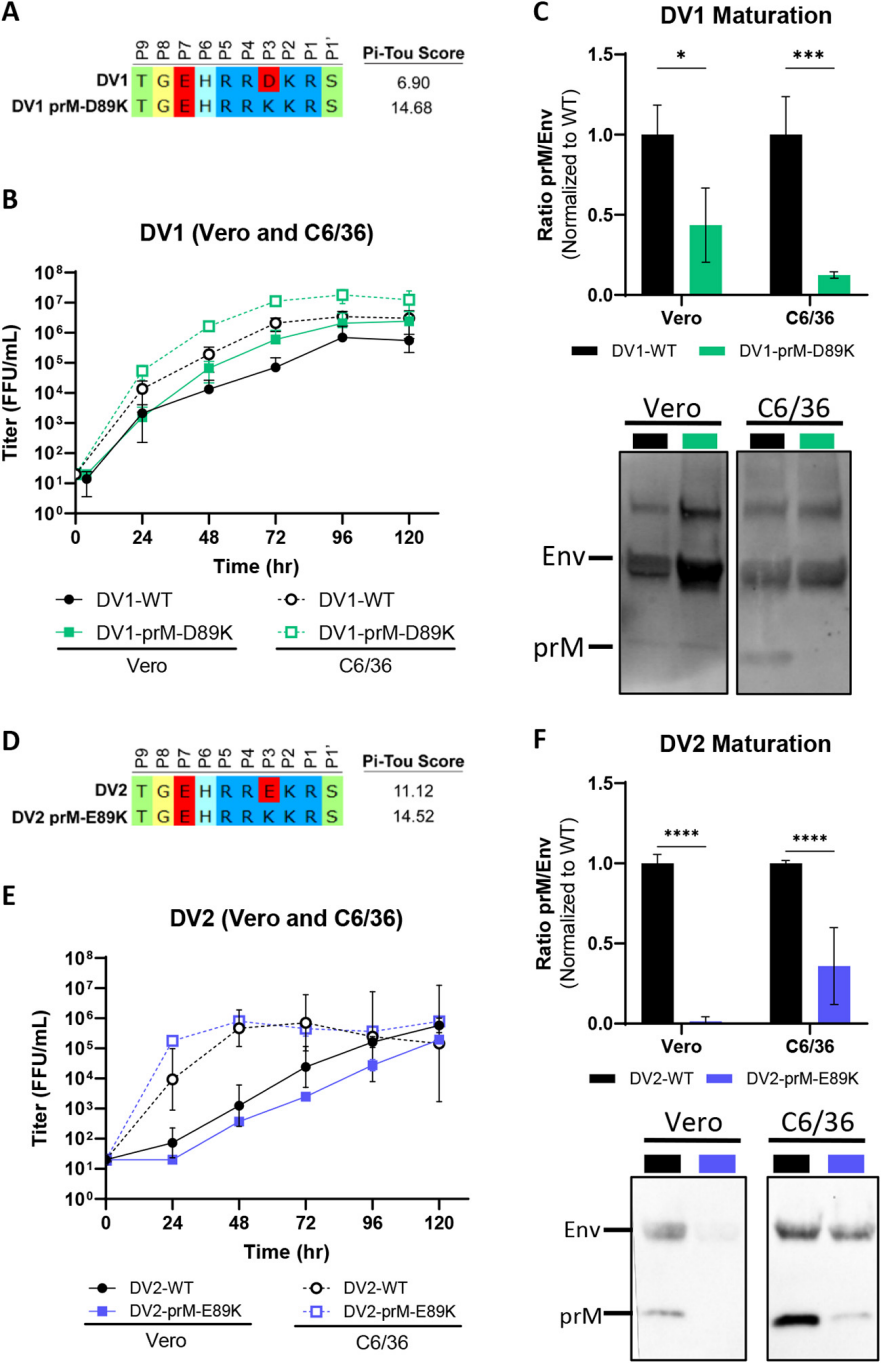
Generation of mature DENV1 and DENV2 via genetic modification. (A) Sequence composition of prM cleavage sites of DV1-WT and DV1-prM-D89K. (B) Growth kinetics of DV1-WT and DV1-prM-D89K in Vero and C6/36 cells. (C) Representative Western blot image (bottom) of DV1-WT and DV1-prM-D89K viral supernatants blotted with anti-Env and anti-prM antibodies, and quantification (top) of viral maturation (prM/Env) normalized to DV1-WT (lower value = more mature). (D) Sequence composition of prM cleavage sites of DV2-WT and DV2-prM-E89K. (E) Growth kinetics of DV2-WT and DV2-prM-E89K in Vero and C6/36 cells. (F) Representative Western blot image (bottom) of DV2-WT and DV2-prM-E89K viral supernatants blotted with anti-Env and anti-prM antibodies, and quantification (top) of viral maturation normalized to DV2-WT. Growth kinetics and maturation of DENV variants were compared to their corresponding wild-type virus using two-way ANOVA multiple comparisons.

As an alternative to ectopic overexpression of furin, genetic optimization of the DENV2 prM furin cleavage site has been shown to enhance DENV maturation independent of cells (31–33). To ensure this type of modification is transferable to all serotypes, we systematically introduced a furin enhancing mutation by substituting a favorable basic lysine (K) residue for an acidic aspartic acid (D) or glutamic acid (E) in the P3 position in all DENV serotypes. Biochemically, the acidic to basic substitution should enhance cleavage by mammalian furin, which is also suggested by an increase in predicted PiTou scores (Fig. 3A). DV1-WT and DV1-prM-D89K displayed no significant difference in virus growth kinetics in Vero (mammalian) and C6/36 (insect) cells (Fig. 3B). In both cell types, DV1-prM-D89K was more mature than DV1-WT, phenocopying the Vero-furin grown DV1-WT (Fig. 3C). In DENV2, while the growth kinetics were similar in insect cells, the DV2-prM-E89K variant displayed slightly reduced growth in mammalian cells compared to DV2-WT. Again, DV2-prM-E89K is more mature than DV2-WT in both mammalian and insect cells.

### Generation of mature DENV3 and DENV4 requires iterative optimization of the prM cleavage sites.

**FIG 4:**
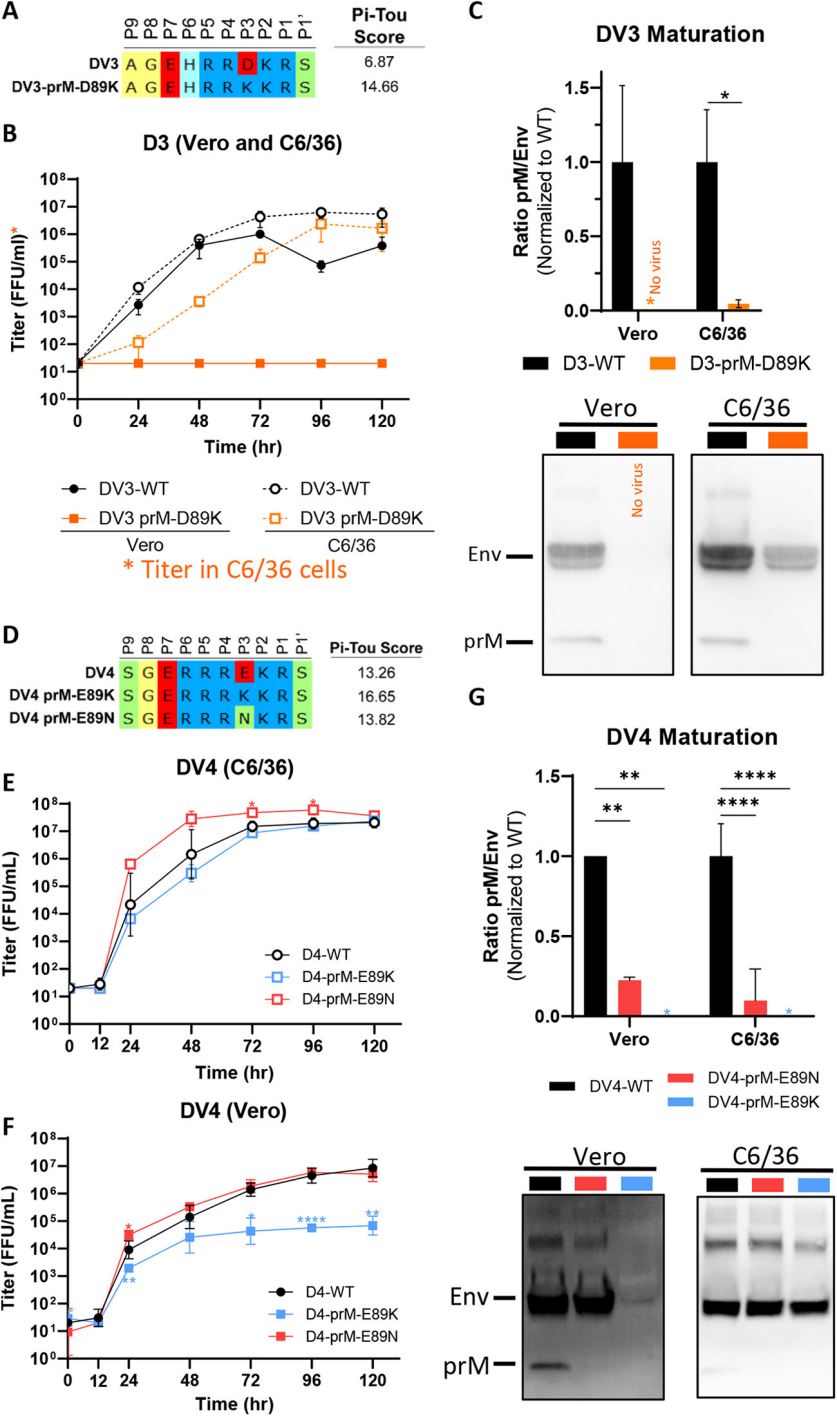
Iterative genetic optimization of mature DENV3 and DENV4. (A) Sequence composition of prM cleavage sites of DV3-WT and DV3-prM-D89K. (B) Growth kinetics of DV3-WT and DV3-prM-D89K in C6/36 and Vero cells. (C) Representative Western blot image (bottom) of DV3-WT and DV3-prM-D89K viral supernatants blotted with anti-Env and anti-prM antibodies and quantification (top) of viral maturation (prM/Env) normalized to DV3-WT. All titers were determined on C6/36 cells. (D) Sequence composition of prM cleavage sites of DV4-WT, DV4-prM-E89K, and DV4-prM-E89N. (E and F) Growth kinetics of DV4-WT, DV4-prM-E89K, and DV4-prM-E89N in C6/36 (E) and Vero (F) cells. (G) Representative Western blot image (bottom) of DV4-WT, DV4-prM-E89K, and DV4-prM-E89N viral supernatants blotted with anti-Env and anti-prM antibodies and quantification (top) of viral maturation (prM/Env) normalized to DV4-WT. Growth kinetics and maturation of DENV variants were compared to their corresponding wild type using two-way ANOVA multiple comparisons.

We introduced a similar acidic to basic mutation on the DV3-WT backbone, generating the isogenic strain DV3-prM-D89K (Fig. 4A). While the growth kinetics between DV3-WT and DV3-prM-D89K are similar in insect cells (Fig. 4B), the DV3-prM-D89K variant is unable to grow on mammalian cells (Fig. 4B). We could not detect any uncleaved prM protein by Western blotting, indicating that DV3-prM-D89K is fully mature (Fig. 4C). Lastly, we introduced the same acidic to basic mutation on the DV4-WT backbone, generating the isogenic strain DV4-prM-E89K (Fig. 4D). Similar to DV3 mutants, the growth kinetics between DV4-WT and DV4-prM-E89K are similar in insect cells (Fig. 4E); however, the DV4-prM-E89K variant displayed a 2-log growth defect compared to DV4-WT on mammalian cells (Fig. 4F). Interestingly, this defect was alleviated when virus was grown at 32°C, suggesting a stability issue of the variant (see Fig. S1a in the supplemental material). A spontaneous mutation, K89N, rapidly emerged in the mutant virus by passage 2 (see Fig. S1b). By the fifth passage, the DV4-prM-E89N variant represented 100% of the viral population (see Fig. S1b), supporting the notion that viruses encoding the E89K mutation were out-competed by the E89N mutation in mammalian cells. We reestablished DV4-prM-E89N via reverse genetics and, as expected, found that DV4-prM-E89N had growth kinetics similar to those of DV4-WT in both Vero and C6/36 cells. The maturations of both variants were tested in comparison to DV4-WT. No prM can be detected in DV4-prM-E89K; due to the low virus yield in mammalian cells, the data suggest that either DV4-prM-E89K is fully mature or the protein input is below detection limit. DV4-prM-E89N is also more mature than DV4-WT in both insect and mammalian cells (Fig. 4G). To look at maturation status more directly, we purified DV4-prM-E89N, DV4-WT, and VF1-grown DV4-WT by sucrose gradient and separated the virions by SDS-PAGE. Confirming the Western blot results, we detected uncleaved prM in DV4-WT grown in Vero cells, indicating partial immature virions. In contrast, no prM band is present in DV4-prM-E89N or in VF1-grown DV4-WT (see Fig. S2).

10.1128/mbio.00386-22.1 FIG S1 (A) Growth kinetics of DV4-WT and DV4-prM-E89K in Vero and C6/36 cells at 32°C (bottom) and 37°C (top). (B) DNA chromatograms of DV4-E89K in C6/36 cells (bottom), as well as Vero cells from early (P2, top) and late (P5, middle) passages. Download FIG S1, TIF file, 0.3 MB. Copyright © 2022 Tse et al. 2022 Tse et al. https://creativecommons.org/licenses/by/4.0/ This content is distributed under the terms of the Creative Commons Attribution 4.0 International license.

10.1128/mbio.00386-22.2 FIG S2 SDS-PAGE of purified DV4-WT, DV4-prM-E89N, and DV4-WT grown in VF1 cells. Viruses were purified and concentrated by two sucrose gradient purifications. Purified samples were tested on stain-free gel and visualized after UV activation. Download FIG S2, TIF file, 0.2 MB. Copyright © 2022 Tse et al. 2022 Tse et al. https://creativecommons.org/licenses/by/4.0/ This content is distributed under the terms of the Creative Commons Attribution 4.0 International license.

Based on the biochemical understanding of mammalian furin, the order of substrate favorability is basic (K or R) > neutral (e.g., N) > acidic (D or E) residues. In DENV3 and DENV4, our result indicates that a highly favorable furin cleavage sites (composed of many basic residues) may negatively impact DENV growth in mammalian cells but not insect cells. Alternatively, the extra lysine residue in DENV3 and DENV4 might pose a specific structural incompatibility in mammalian cells. Nevertheless, at least two rounds of iterative optimization were needed to generate genetically mature DENV4.

### Directed evolution reveals high levels of plasticity in the DENV2 prM cleavage site.

**FIG 5:**
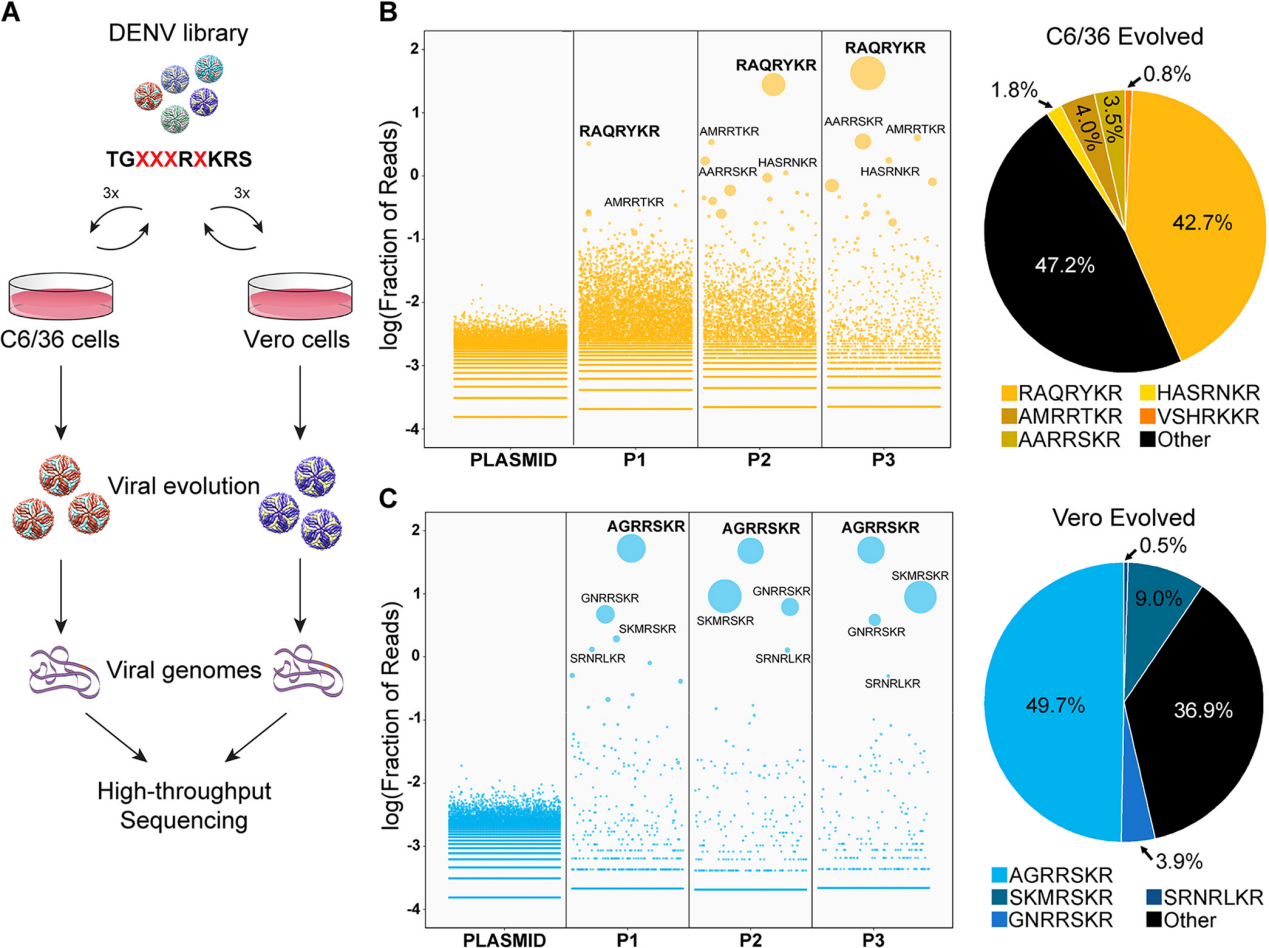
Directed evolution of DENV2 prM cleavage site in Vero and C6/36 cells. (A) Schematic of directed evolution from library generation to high-throughput sequencing. (B and C) Enrichment plots of prM cleavage site sequences from plasmid library to viral population at the third passage (P3) and the proportion, as well as sequence of the top 5 enriched sequences in C6/36 cells (yellow) (B) and Vero cells (cyan) (C).

Our results with DENV3 and DENV4 indicate that the furin cleavage site may play additional role in viral fitness independent of maturation. Therefore, there may exist optimal sequences of the prM cleavage site for efficient *in vitro* growth, as well as maturation. To test this hypothesis, we selected DV2, the most immature DENV in our panel, which also had reduced viral fitness upon genetic alteration. We performed saturation mutagenesis and directed evolution to simultaneously screen thousands of DENV2 prM cleavage site variants for efficient growth in mammalian and insect cultures. To ensure the viability of the library, the mutation sites were designed to preserve the minimal core of furin cleavage site, R-X-K-R (Fig. 5A). We selected the P3, P5, P6, and P7 positions of the prM cleavage site for saturation mutagenesis. The viral library was generated and passaged three times in either insect or mammalian cells, and each passage of the virus were deep sequenced along with the plasmid library (Fig. 5A). The theoretical amino acid diversity of the library approached 160,000 variants (excluding stop codons), which was fully represented in the plasmid library (Table 2). As expected, viral diversity rapidly dropped after one passage, to 0.7% (1,148 unique variants) and 16.2% (25,942 unique variants) of the theoretical maximum in mammalian and insect cells, respectively, and further diminished in subsequent passages (Table 2). The relatively large number of viable DENV2 variants in both cells indicated a high degree of plasticity within the prM cleavage site of DENV (Table 2). Interestingly, insect cells were more tolerant to prM cleavage site variations than mammalian cells, with 10 times more unique viable variants at the end of passage 3 (Table 2), suggesting a greater range of plasticity in the prM cleavage site.

**TABLE 2:** Summary of plasmids and passages diversities of DV2 directed evolution

Plasmid or passage (P)	C6/36 evolved		Vero evolved	
	No. of unique sequences	% maximum	No. of unique sequences	% maximum
Plasmid	164,569	102.86*	164,569	102.86*
P1	25,942	16.21	1,148	0.72
P2	14,119	8.82	719	0.45
P3	6,026	3.77	683	0.43

*, Including stop codons.

After three rounds of selection, two different dominant variants, TGRAQRYKR|S (DV2-C1, from insect cells) and TGAGRRSKR|S (DV2-V1, from mammalian cells), emerged, each representing almost 50% of their respective viral populations (Fig. 5B). Neither variant contained an acidic residue in the proximity (P1 to P7) of the furin cleavage site, as opposed to two acidic residues in the WT sequence (Fig. 5B and C). We analyzed the top 50 selected variants from both insect and mammalian cells, and out of 100 variants only four contained an acidic residue (Table 3), showing a general preference to exclude acidic residues at the furin cleavage site in both cell types. Interestingly, there is only one sequence shared between the top 50 variants evolved from insect and mammalian cells after three passages, suggesting host specificity. Although founder effects cannot be excluded in directed-evolution experiments, the clear deselection of acidic residues and the distinct populations found in insect and mammalian cells despite the shared starting library highlight both the shared and differential selective pressures exerted by the two hosts.

**TABLE 3:** Top 50 enriched sequences and PiTou scores of DV2 directed evolution

Name	Sequence (5′–3′)	PiTou score
DENV2-C1	TGRAQRYKRS	7.33832
DENV2-C2	TGAARRSKRS	14.843
DENV2-C3	TGAAVRSKRS	12.3488
DENV2-C4	TGAMRRTKRS	11.6874
DENV2-C5	TGRIQRLKRS	3.4267
DENV2-C6	TGNSGRHKRS	11.4735
DENV2-C7	TGFSTRNKRS	10.0498
DENV2-C8	TGAANRVKRS	11.1661
DENV2-C9	TGSVQRIKRS	8.36797
DENV2-C10	TGVSRRSKRS	15.3795
DENV2-C11	TGSTRRDKRS	7.72902
DENV2-C12	TGTKGRVKRS	12.3958
DENV2-C13	TGTTHRHKRS	11.0362
DENV2-C14	TGLPVRSKRS	12.6207
DENV2-C15	TGSRTRSKRS	13.0648
DENV2-C16	TGSTRRHKRS	13.4014
DENV2-C17	TGHVSRSKRS	12.2249
DENV2-C18	TGTRNRKKRS	13.8726
DENV2-C19	TGFTNRVKRS	11.247
DENV2-C20	TGSNSRSKRS	12.156
DENV2-C21	TGASSRHKRS	12.598
DENV2-C22	TGQVHRSKRS	11.0663
DENV2-C23	TGTAKRSKRS	13.7489
DENV2-C24	TGNLRRTKRS	14.5679
DENV2-C25	TGFSSRSKRS	14.164
DENV2-C26	TGGKVRNKRS	10.82
DENV2-C27	TGGHVRHKRS	10.4826
DENV2-C28	TGSAQRSKRS	11.1173
DENV2-C29	TGEKKRAKRS	11.8098
DENV2-C30	TGITTRSKRS	11.6576
DENV2-C31	TGAHKREKRS	10.4483
DENV2-C32	TGGARRQKRS	14.399
DENV2-C33	TGLTRRSKRS	14.9674
DENV2-C34	TGASRRAKRS	13.0953
DENV2-C35	TGHMTRAKRS	6.18586
DENV2-C36	TGLKVRHKRS	11.5383
DENV2-C37	TGGAKRGKRS	10.9028
DENV2-C38	TGHASRNKRS	11.3823
DENV2-C39	TGGDARRKRS	9.84567
DENV2-C40	TGVASRTKRS	13.846
DENV2-C41	TGNYPRNKRS	7.18517
DENV2-C42	TGERTRSKRS	13.0648
DENV2-C43	TGRRMRSKRS	11.7847
DENV2-C44	TGERKRAKRS	12.2477
DENV2-C45	TGMNKRSKRS	12.312
DENV2-C46	TGHPSRGKRS	11.0941
DENV2-C47	TGVRARTKRS	13.9117
DENV2-C48	TGRSLRSKRS	12.3732
DENV2-C49	TGMPRRSKRS	15.082
DENV2-C50	TGRAVRHKRS	10.7828
DENV2-V1	TGAGRRSKRS	14.843
DENV2-V2	TGGNRRSKRS	13.4061
DENV2-V3	TGSKMRSKRS	11.3468
DENV2-V4	TGSRNRLKRS	12.4246
DENV2-V5	TGMAKRSKRS	13.7489
DENV2-V6	TGTAKRSKRS	13.7489
DENV2-V7	TGYRQRSKRS	12.3466
DENV2-V8	TGLSRRSKRS	15.3795
DENV2-V9	TGGFRRSKRS	11.508
DENV2-V10	TGRQARSKRS	11.0661
DENV2-V11	TGKMRREKRS	8.66191
DENV2-V12	TGSNKRHKRS	10.7461
DENV2-V13	TGRTGRTKRS	12.8044
DENV2-V14	TGERARVKRS	12.8337
DENV2-V15	TGRYKRDKRS	4.10841
DENV2-V16	TGAGRSSKRS	−4.9918
DENV2-V17	TGAWRRSKRS	−5.18386
DENV2-V18	TGAGRRRKRS	15.0224
DENV2-V19	TGGKSRVKRS	13.5558
DENV2-V20	TGRPVRSKRS	12.6207
DENV2-V21	TGHSRREKRS	12.5334
DENV2-V22	TGWGKRSKRS	13.7489
DENV2-V23	TGTGRRMKRS	12.5588
DENV2-V24	TGAGRRIKRS	13.5353
DENV2-V25	TGRSKRSKRS	14.2854
DENV2-V26	TGSVRRVKRS	12.5076
DENV2-V27	TGASHRSKRS	13.016
DENV2-V28	TGMSKRTKRS	14.4649
DENV2-V29	TGAGRRNKRS	12.5588
DENV2-V30	TGPGRRSKRS	14.843
DENV2-V31	TGFKHRVKRS	12.2057
DENV2-V32	TGGRHRNKRS	11.2579
DENV2-V33	TGAGSRSKRS	13.6629
DENV2-V34	TGAGLRSKRS	11.8703
DENV2-V35	TGAARRSKRS	14.843
DENV2-V36	TGAGRRTKRS	15.0224
DENV2-V37	TGAERRSKRS	11.508
DENV2-V38	TGSKLRSKRS	12.6269
DENV2-V39	TGAVRRSKRS	13.4061
DENV2-V40	TGTGRRSKRS	14.843
DENV2-V41	TGARRRSKRS	15.626
DENV2-V42	TGATKRSKRS	13.8734
DENV2-V43	TGDGRRSKRS	14.843
DENV2-V44	TGSKIRTKRS	13.2837
DENV2-V45	TGAGCRSKRS	−5.14866
DENV2-V46	TGSGRRSKRS	14.843
DENV2-V47	TGGTRRSKRS	14.9674
DENV2-V48	TGVGRRSKRS	14.843
DENV2-V49	TGISKRGKRS	11.1775
DENV2-V50	TGSRNRFKRS	11.0389

### Direct-evolved DENV2 variants are highly mature and produce higher yield.

**FIG 6:**
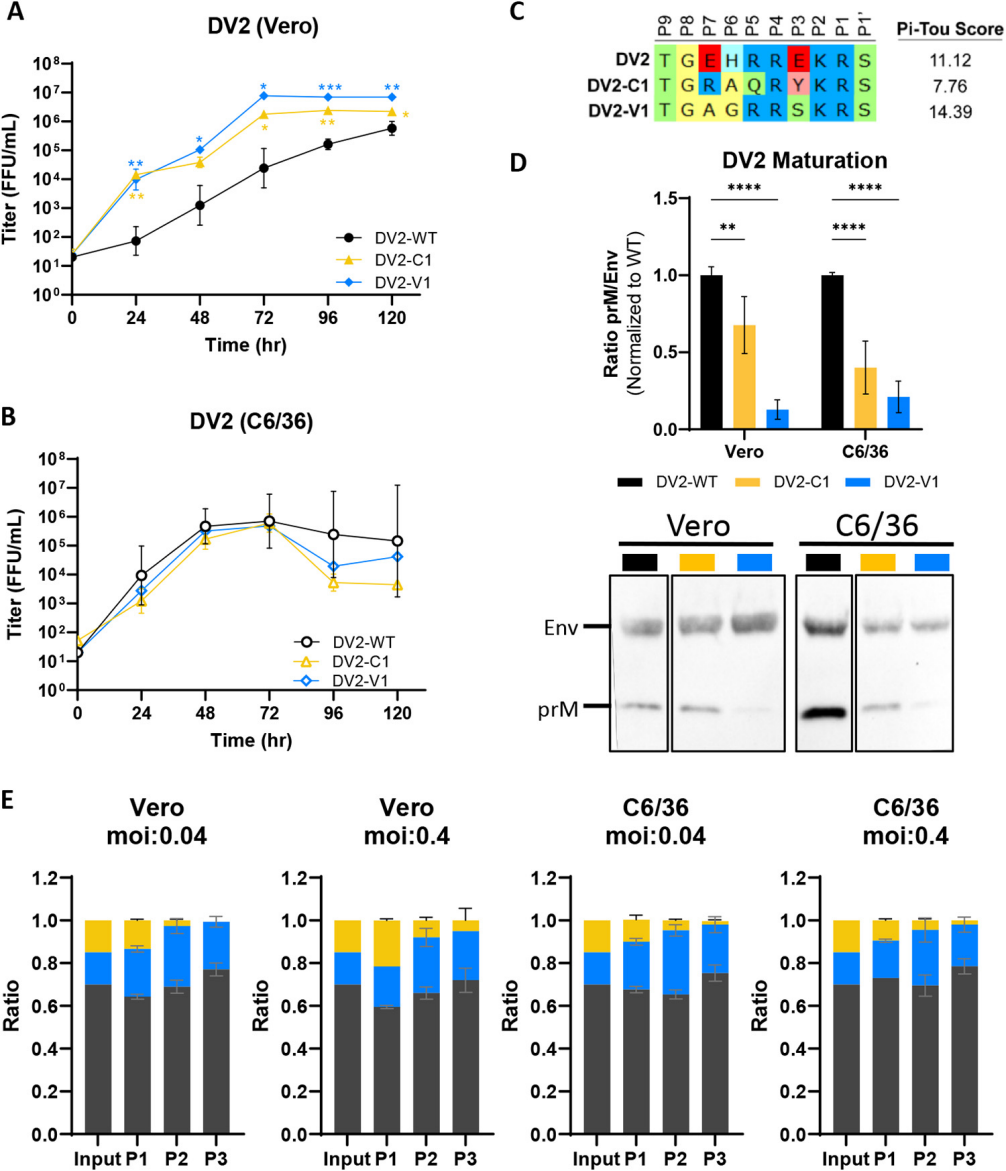
Replicative fitness and maturation of evolved DV2 variants. (A and B) Growth kinetics of DV2-WT, DV2-C1, and DV2-V1 in Vero (A) and C6/36 (B) cells. (C) Sequence composition of prM cleavage sites of DV2-WT, DV2-C1, and DV2-V1. (D) Representative Western blot image (bottom) of DV2-WT, DV2-C1, and DV2-V1 viral supernatants blotted with anti-Env and anti-prM antibodies, and quantification (top) of viral maturation (prM/Env) normalized to DV2-WT (lower value = more mature). (E) Competition assay of DV2-WT, DV2-C1, and DV2-V1 at equal FFU ratio in Vero and C6/36 cells at high and low MOIs. Viral genomes were quantified by digital droplet PCR (ddPCR) multiplexed with a general DV2 probe and strain-specific probes. Growth kinetics and maturation of DV2 variants were compared to DV2-WT using two-way ANOVA multiple comparisons.

The top evolved variants, DV2-V1 and DV2-C1, were rederived via reverse genetics for further characterization. Both DV2-V1 and DV2-C1 grow to higher titer than DV2-WT in Vero cells, although we observed a slight nonsignificant drop in titer in C6/36 cells at 96 to 120 h postinfection (hpi) (Fig. 6A and B). When we tested maturation status, we found that DV2-V1 is almost fully mature, while DV2-C1 is only 30% more mature than DV2-WT when grown in Vero cells (Fig. 6D). When the viruses are grown in C6/36, both variants are 60 to 70% more mature than DV2-WT (Fig. 6D). To determine the relative fitness of these variants to DV2-WT, we performed competition assays of all three viruses on both Vero and C6/36 cells at low (0.04) and high (0.4) multiplicities of infection (MOIs). Although we mixed equal focus-forming units (FFU) of each virus as input, the RNA genome ratio of WT to V1 to C1 is 4.6:1:1, suggesting a different particle to PFU ratio in DV2-WT (Fig. 6E). After three consecutive passages, DV2-C1 was out competed by DV2-WT and DV2-V1 under all conditions tested (Fig. 6E). In contrast, DV2-V1 was able to maintain its population after three passages, displaying similar replicative fitness to DV2-WT (Fig. 6E).

### Impact of maturation status on DENV epitope presentation and antigenicity.

**FIG 7:**
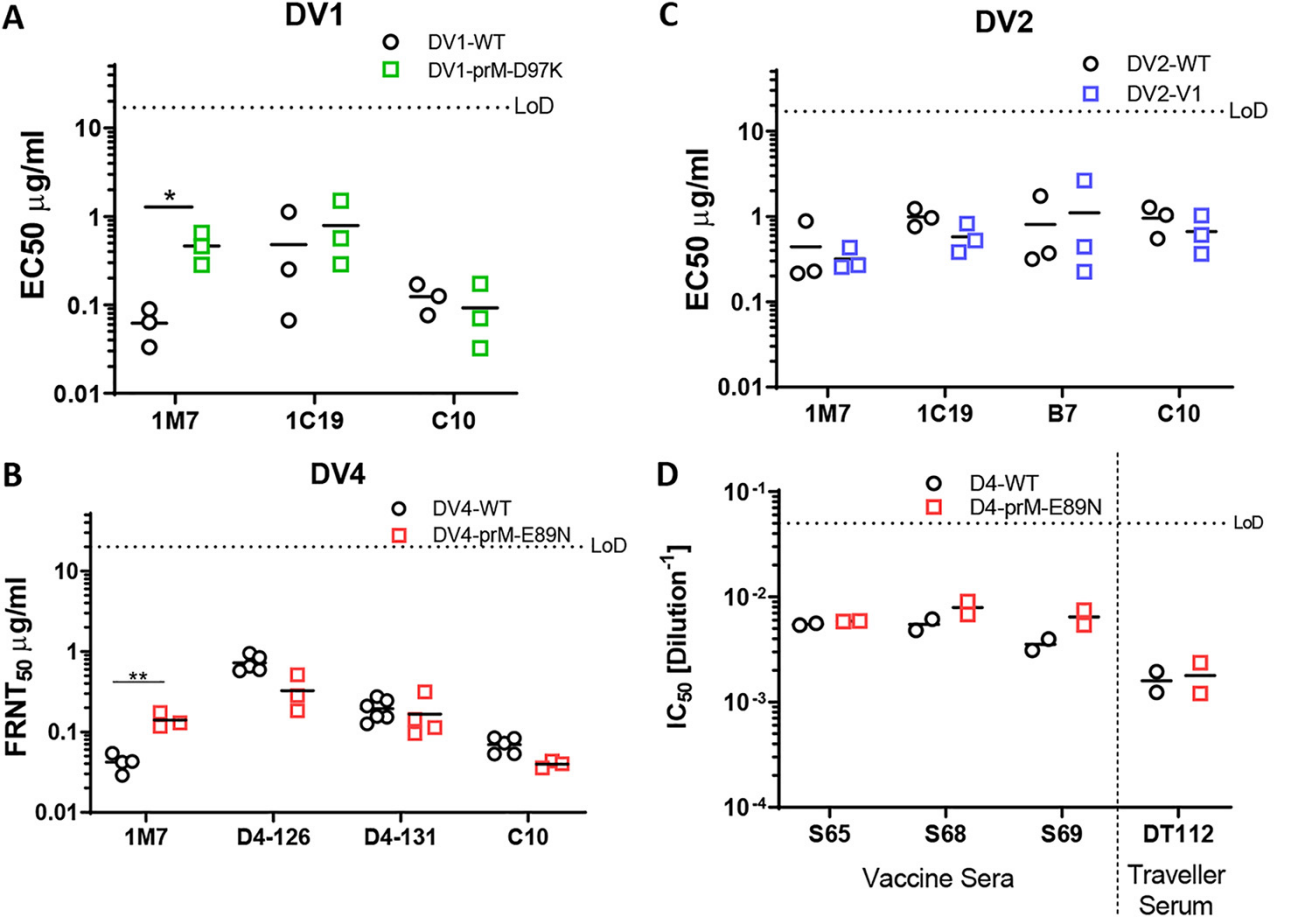
Antigenic profile of mature DENV. (A) FRNT_50_ of DV1-WT and DV1-prM-D89K against 1C19, EDE1-C10 (C10), and 1M7. (B) FRNT_50_ of DV4-WT and DV4-prM-E89N against D4-126, D4-131, C10, and 1M7. (C) FRNT_50_ of DV2-WT and DV2-V1 against 1C19, EDE2-B7 (B7), C10, and 1M7. (D) FRNT_50_ of vaccine sera and traveler serum against DV4 and DV4-prM-E89N.

Now that we had created a set of genetically mature DENV viruses, we next evaluated the impact of maturation status on antigenicity. We selected several monoclonal antibodies (MAbs) targeting different regions of the DENV E glycoprotein, including C10 (Envelope-Dimer-Epitope 1) (34), B7 (Envelope-Dimer-Epitope 2) (34), 1C19 (BC loop) (35), and 1M7 (fusion loop) (35). As expected, antibody epitopes that are not maturation dependent are preserved, as evidenced by antibodies such as C10, B7, and 1C19 which showed no difference in Focus Reduction Neutralization Titer 50 values (FRNT_50_) (Fig. 7A to C). However, the fusion loop targeting antibody 1M7 showed significantly different FRNT_50_ values between fully mature and less mature DENVs in DENV1 and DENV4, but not in DENV2 (Fig. 7A to C). For DENV4, we also tested polyclonal sera from patients 180 days after DENV4 vaccination or from naturally infected patients from a traveler cohort. Polyclonal serum contains a mixture of antibodies which may or may not be affected by virion maturation status. Unsurprisingly, the FRNT_50_ of polyclonal serum was equivalent for fully mature and partially mature DENV4 (Fig. 7D).

## DISCUSSION

In this report, we provided both cell-based and genetics-based methods to produce fully mature DENVs and demonstrated the ability to evolve highly fit and mature DENV variants via a single round saturation mutagenesis of the prM furin cleavage site. Although biochemical understanding of mammalian furin and algorithms such as PiTou (used here) and ProP could be used as a reference to rationally design optimal furin cleavage site (29, 36), it must be noted that all predictions are based on mammalian furin rather than invertebrate furin proteases. Human furin and Aedes aegypti furin-like-proteases only share 39% sequence identity (see Fig. S3) and *Drosophila* furin has been shown to have different substrate preferences (23). This partially explains why PiTou predictions do not correlate well with the more complex DENV maturation or other viruses that cycle between vertebrate and in vertebrate hosts. Other reasons include cleavage site accessibility, protein structure, stability, and the stem region of prM, which can affect maturation in DENV and other viruses (37–39). Indeed, the degree of maturation differs among DENV genotypes with identical prM proteins, indicating contributions by an envelope-dependent maturation determinant as well (40). Due to the complicated nature of furin cleavage and maturation in DENV, rational design of furin cleavage site is a process of trial and error. Previous studies mutated the furin cleavage site to generate mature DENV2, as well as dengue virus-like particles (41, 42). However, the replication fitness of the resulting mutants was compromised (32, 33). Here, we engineered compatible furin cleavage sequences for all four DENV serotypes which generate mature DENV variants with nearly identical replication fitness.

10.1128/mbio.00386-22.3 FIG S3 Amino acid sequence identity matrix of furin and furin-like proteases between humans and Aedes aegypti. Download FIG S3, TIF file, 0.3 MB. Copyright © 2022 Tse et al. 2022 Tse et al. https://creativecommons.org/licenses/by/4.0/ This content is distributed under the terms of the Creative Commons Attribution 4.0 International license.

Substitution of the acidic (D or E) P3 residues with basic (K) residues increased furin cleavage efficiency and resulted in fully mature and replication-competent DENV1 and -2. However, the same acidic to basic substitution is not compatible in DENV 3 and 4, instead resulting in viruses with large growth defect on mammalian cells, which were alleviated when grown at 32°C. In contrast, this growth defect was not observed in DENV grown in VF1 cells, excluding the possibility that fully mature DENV3 and -4 are less fit at 37°C. Possible explanations include impacts on RNA or protein stability/folding, which could be alleviated at a lower temperature (13). These results hint that the unfavorable acidic residues at P3 may play a structural or regulatory role in DENV fitness. A spontaneous mutation (K89N) in DENV4 restored viral fitness, generating a mature and replication competent DENV4. However, the complete abrogation of viral growth seen in DV3-prM-D98K did not allow for spontaneous mutation. Overall, such stepwise optimization of the furin cleavage site is time-consuming, labor-intensive, and not guaranteed to be successful.

Using directed evolution, we tested the fitness of thousands of DENV2 prM cleavage site variants at once and selected for compatible sequences that do not compromise replication fitness. Importantly, due to the stochastic nature of directed-evolution experiment, our variants were not expected to be the only optimal sequences among the 160,000 variants tested; equally optimal sequences could be identified in a different experimental setup. Our data revealed (i) a high sequence plasticity of the furin cleavage site, especially in insect cells; (ii) that a more mature DENV is favorable in both cell types; and (iii) that insect and mammalian cells exert specific selective pressure. We observed 10 times more viable variants in insect cells compared to mammalian cells, indicating insects have higher tolerance of mutation in furin sites. This could be intrinsic to the innate immunity and substrate preference of insect vitellogenin/furin, or due to the reduced growth temperature of insect cells at 32°C. Such a high tolerance of mutation in insect cells could drive viral diversity and emergence in nature.

In addition, we observed that top Vero- and C6/36-selected variants excluded acidic resides in the cleavage site and are more mature. The selected variants also displayed enhanced growth kinetics as well as a slight increase in peak titer in Vero cells. Our result suggest that experimental evolution specifically selects for more mature DENVs, indicating that a similar strategy can be apply to other serotypes to generate genetically determined mature DENVs. Evolutionarily, despite high experimental plasticity, DENV furin cleavage sites are extremely conserved in nature with the presence of two acidic residues. We speculate deselection of acidic residues at the cleavage site could be tissue culture adaptations, and the growth advantage may not be reflected in natural infections or *in vivo*. Indeed, our competition assays revealed no fitness advantage for the evolved variants. This discrepancy between our experimental evolution and observations in nature suggests either an unknown advantage of the WT cleavage site or a bottleneck effect in nature. It is of interest to conduct future experiments focused on *in vivo* evolution of the viral library using Aedes aegypti mosquitoes.

Only one furin cleavage site sequence is shared in the top 50 selected variants from both hosts, even though insect cells supported 10 times more variants. This cannot be explained by the sporadic nature of experimental evolution; in fact, this strongly supports differential selective pressure being exerted by the two hosts, which warrants further investigation. Interestingly, the mammalian selected variant, DV2-V1, out competed the insect selected variant, DV2-C1, on both Vero and C6/36 cells. We can speculate a model wherein the mosquito life cycle allows diversification and/or antigenic drift of DENV, which requires further adaptation in mammalian hosts. In reverse, the mammalian adapted virus can readily infect the mosquito host and further diversify to evade mammalian adaptive immunity, creating a cycle of virus evolution. This model is supported by the diverse populations and sylvatic strains of DENV, which are found in mosquitos (43). Future directed-evolution experiments of other regions of DENV will increase our understanding of DENV evolution between hosts.

Against antibodies targeting maturation independent epitopes (such as EDE1, EDE2, and BC loop epitopes), our genetically modified mature DENVs show neutralization profiles similar to those of the wild type, suggesting mutations at the prM cleavage site do not affect the overall viral protein structure. However, maturation-dependent epitopes present only in one form of the virus, such as the fusion loop (17), show a different neutralization profile against our mature DENVs except DENV2. DENV2 has been shown to be very flexible, possibly accounting for the insensitivity of 1M7 neutralization to maturation status (44, 45). Our results support earlier studies showing differences in antigenicity between mature and immature DENVs using furin overexpression cells (13), while providing new opportunities for studying the role of maturation in antigenicity, vaccine design, and *in vivo* replication and pathogenesis. Recent studies have demonstrated a potential disconnect between neutralizing antibody correlates of protective immunity in vaccine recipients (46). Our data are consistent with earlier studies showing that the maturation status of DENV particles could have major implications for neutralization assay outcomes and result in bias during the determination of correlates of protection for vaccine studies (12, 17, 47). These findings reinforce the importance of monitoring DENV maturation status in vaccine development, and our engineered strains provide a universal way to control DENV maturation for live-attenuated vaccine candidates independent of cell and host.

Like many studies, our report generated additional questions. Biologically, does DENV maturation play a more critical role than simply preventing premature fusion during production? Could maturation also play a role in vector-to-host or host-to-vector transmission? Is fully mature DENV advantageous or deleterious in mosquitoes and mammals? What determinants outside of the primary cleavage site sequence regulate maturation efficiency? Will biologically stabilized virions drive selection of unique subsets of neutralizing antibodies after infection? Clinically, the antigenic differences between mature and immature DENV require more comprehensive investigation. Furthermore, a new class of vaccines could be imagined based on stabilized mature particles which elicit maturation discriminatory antibodies. Given the clinical relevance and evolutionary relationship of DENV maturation, our study adds to understanding of DENV maturation control and provides essential tools for future investigations.

## MATERIALS AND METHODS

### Cells, plasmids, and viruses.

Mosquito (Aedes albopictus) C6/36 cells (ATCC CRL-1660) were maintained in minimum essential medium (MEM; Gibco) supplemented with 5% fetal bovine serum (FBS; HyClone), 100 U/mL penicillin and 100 mg/mL penicillin/streptomycin (P/S; Gibco), 0.1 mM nonessential amino acids (NEAA; Gibco), HEPES (Gibco), and 2 mM GlutaMAX (Gibco), followed by incubation in the presence of 5% CO_2_ at 32°C. Vero cells (ATCC CCL-81) and VF-Hi and VF-Lo cells (generated from this study) were maintained in Dulbecco modified Eagle medium (DMEM; Gibco) supplemented with 10% FBS, P/S, NEAA, and HEPES and incubate in 5% CO_2_ at 37°C. DENV variants were generated by site-directed mutagenesis using Q5 high-fidelity DNA polymerase (NEB), followed by DENV reverse genetics (see below). The Env and prM of all DENV variants were sequence confirmed. DV1, -2, and -3 and 4-WT viruses were grown in C6/36 or Vero cells maintained in infection media. C6/36 infection media contain Opti-MEM (Gibco) supplemented with 2% FBS, 1% P/S, 0.1 mM NEAA, 1% HEPES, and 2 mM GlutaMAX. Vero infection medium is the same as the growth medium except with a 2% FBS supplement.

### DENV reverse genetics.

Recombinant viruses were constructed using a four-plasmid cloning strategy as described previously (48). The DENV genome was divided into four fragments (A to D) and subcloned into four separate plasmids. A T7 promoter was introduced into the 5′ end of the A fragment, and unique type IIS restriction endonuclease cleavage sites are introduced into the 5′ and 3′ ends of each fragment to allow for systematic assembly into a full-length cDNA, from which the full-length RNA transcripts can be derived. Plasmid DNA was grown in Top10 chemical component cells (Thermo Fisher), digested with the corresponding enzymes, gel purified, and ligated together with T4 DNA ligase (NEB). Ligation products were purified by chloroform extraction. The purified ligation product was used as a template for *in vitro* transcription to generate infectious genome-length capped viral RNA transcripts using T7 RNA polymerase (Thermo Fisher). RNA was electroporated into either C6/36 or Vero cells. Cell culture supernatant containing virus was harvested 4 to 5 days postelectroporation as passage zero. During the subsequent passages following infection, the cells were grown in infection media.

### Stable cell line generation, VeroFurin-Clone-1 (VF1).

Human furin was cloned in the sleeping beauty transposon plasmid (30) pSB-bi-RP (Addgene, catalog no. 60513), transfected along with transposase and pCMV(CAT)T7-SB100 (Addgene, catalog no. 34879) into Vero cell using PEI Max (MW 40,000; Polysciences), and selected with 2.5 μg/mL Puromycin (Gibco). Clonal cell lines were generated through limited dilution of the polyclonal cell line on a 96-well plate at the concentration of 0.3 cell/well.

### DENV growth kinetics and quantification.

A total of 500,000 Vero or C6/36 cells were seeded in each well of a six-well plate 1 day prior infection. Cells were infected with DENV at an MOI of 0.05 to 0.1, assuming 1 × 10^6^ cells on the day of infection. Cells were washed three times with PBS and replenished with 3 mL of infection medium after 1 h of inoculation at 37°C in 5% CO_2_ incubator. Then, 300 μL of viral supernatant was collected; fresh medium was replenished at 0, 24, 48, 72, 96, and 120 hpi; and the samples were stored at −80°C. Titers of the viral supernatant were determined using a standard DENV focus-forming assay. In brief, Vero cells were seeded at 2 × 10^4^ cells/well in a 96-well plate. Next, 50 μL of serial diluted viral supernatant was added to each well, followed by incubation for 1 h at 37°C in a 5% CO_2_ incubator. Finally, 125 μL of overlay (Opti-MEM + 5% methyl cellulose + NEAA + P/S) was added to each well, followed by incubation for 48 h at 37°C + 5% CO_2_. Each well was rinsed three times with PBS and fixed with 10% formalin in PBS for staining. Vero cells were blocked in permeabilization buffer (eBioscience) with 5% nonfat dried milk. Two primary antibodies, anti-prM MAb 2H2 and anti-Env MAb 4G2, from nonpurified hybridoma supernatant were used at a 1:500 dilution in blocking buffer. Goat anti-mouse secondary conjugated with horseradish peroxidase (HRP; SeraCare’s KPL) were diluted at 1:1,000 in blocking buffer. Foci were developed using TrueBlue HRP substrate (SeraCare’s KPL) and counted using an automated Immunospot analyzer (Cellular Technology, Ltd.). All experiments were performed independently at least three times.

### Immunostaining and Western blotting for human furin.

Cells were fixed in 10% formalin in phosphate-buffered saline (PBS) and permeabilized with permeabilization buffer (eBioscience). Rabbit anti-furin (Thermo, PA1-062, 1:1,000) was used as primary antibody. Goat anti-rabbit Alexa 488 (Invitrogen, 1:2,000) as a secondary antibody. For Western blotting, cells were lysed in 1% Triton X-100, 100 mM Tris, 2 M NaCl, and 100 mM EDTA. Cell lysates were subjected to SDS-PAGE and blotted onto a polyvinylidene difluoride membrane. Furin bands were detected using rabbit anti-furin polyclonal at 1:1,000, and goat anti-rabbit HRP (Invitrogen, 1:5,000) was used as a secondary antibody.

### Western blotting for DENV maturation.

Viral stocks or supernatant from DENV growth curves at 120 hpi were diluted with 4× Laemmli Sample Buffer (Bio-Rad) and boiled at 95°C for 5 min. After SDS-PAGE electrophoresis, proteins were transferred to polyvinylidene difluoride membrane and blocked in blocking buffer consist of 3% nonfat milk in PBS + 0.05% Tween 20 (PBS-T). The membrane was incubated with polyclonal rabbit anti-prM (1:1,000; Invitrogen, catalog no. PA5-34966) and purified human anti-Env (fusion loop) 1M7 (2 μg/mL) in 2% bovine serum albumin + PBS-T solution for 1 h at 37°C. The primary antigen-antibody complex was detected by incubating the blot with goat anti-rabbit IgG-HRP (1:10,000; Jackson-ImmunoLab) and sheep anti-human IgG HRP (1:5,000, GE Healthcare) in 3% milk in PBS-T, for 1 h at room temperature. Membranes were developed by SuperSignal West Pico Plus chemiluminescent substrate (Thermo Fisher). Western blot images were captured with an iBright FL1500 imaging system (Invitrogen). The pixel intensity of individual bands was measured using ImageJ, and the relative maturation was calculated by using the following equation: (prM_Exp_/Env_Exp_)/(prM_WT_/Env_WT_). All experiments were performed independently a minimum of three times.

### FRNT assay.

Focus reduction neutralization titer assay (FRNT) assays were performed on Vero cells, as described previously (49). Briefly, 2 × 10^4^ Vero cells were seeded in a 96-well plate. Antiserum or MAbs were serially diluted and mixed with DENVs (80 to 100 FFU/well) at a 1:1 volume ratio, followed by incubation at 37°C for 1 h without the cells. The mixture was transferred to the 96-well plate with Vero cells and incubated at 37°C for 1 h. The plate is subsequently overlaid with overlay medium (see above). Viral foci were stained and counted, as described above. Data were fitted with variable slope sigmoidal dose-response curves, and FRNT_50_ values were calculated with top or bottom restraints of 100 and 0, respectively. All experiments were performed independently at least two times, due to limited amounts of human serum.

### DENV2 library generation and directed evolution.

DENV prM libraries were engineered through saturation mutagenesis on amino acid residues P3, P5, P6, and P7 of the DENV furin cleavage site based on previously published protocol (50). In brief, degenerate NNK oligonucleotides (Integrated DNA Technologies) were used to amplify the prM region to generate a library with mutated prM DNA fragments. To limit bias and ensure accuracy, Q5 high-fidelity polymerase (NEB) was used and limited to <18 cycles of amplification. The DNA library was cloned into the DENV reverse-genetics system plasmid A to create a plasmid library by standard restriction digestion. Ligation reactions were then concentrated and purified by ethanol precipitation. Purified ligation products were electroporated into DH10B ElectroMax cells (Invitrogen) and directly plated on multiple 5,245-mm^2^ bioassay dishes (Corning) to avoid bias from bacterial suspension cultures. Colonies were pooled and purified using a Maxiprep kit (Qiagen). The plasmid library was used for DENV reverse genetics as described above. The *in vitro*-transcribed DENV RNA library was electroporated in either Vero or C6/36 cells, and the viral supernatants were passaged three times every 4 to 5 days in the corresponding cells for enrichment.

### High-throughput sequencing and analysis.

Viral RNA was isolated using a QIAamp Viral RNA minikit (Qiagen). Amplicons containing the library regions were prepared for sequencing through two rounds of PCR, using the Illumina TruSeq system and Q5 Hot Start DNA polymerase (NEB). Primers for the first round of PCR were specific to the DENV2 prM sequence with overhangs for Illumina adapters. This PCR product was purified and used as a template for a second round of PCR using the standard Illumina P5 and P7 primers with barcodes and sequencing adaptors. PCR products were purified and analyzed on a Qubit 4 fluorometer (Invitrogen) and Bioanalyzer (Agilent Technologies) for quality control. Amplicon libraries were diluted to 4 nM and pooled for sequencing, which was carried out on a MiSeq system with 300-bp paired-end reads. Plasmid and P0 libraries were sequenced at a depth of ~1 million reads per sample; further passages were sequenced with depth between 300,000 to 1 million reads to sample. A custom perl script (50) was used to analyze the sequences, and custom R scripts were used to plot the data.

### DENV competition assay.

DV2-WT, DV2-V1, and DV2-C1 were mixed at 1:1:1 FFU ratio and used to infect Vero or C6/36 cells at two different MOIs (0.4 or 0.04) in a six-well plate. At 4 dpi, supernatants were collected, and titers were determined before the next round of infection for a total of three passages. Viral RNA was isolated from each passage using the Maxwell RSC48 (Promega) automated RNA extraction machine. Viral cDNA was generated using SuperScript IV reverse transcriptase (Invitrogen) using universal reverse primer 5′-CTRATYTCCATSCCRTACCAGC-3′. To quantify the relative amount of each variant in the population, we developed a multiplex assay using digital droplet PCR (ddPCR) using general DENV2 probe (HEX) and specific cleavage site probes (FAM) (Table 4).

**TABLE 4:** Primers and probes used for DENV competition assay

Primer or probe	Sequence (5′–3′)
DV2-C1	
DV2-C1-F	GTAACTTATGGGACTTGTACTACCAC
DV2-C1-Probe-FAM	AGCGCTTATATCTCTGAGCACGTCC
DV2-C1-R	CACATGTGGAACGAGTGC
	
DV2-V1	
DV2-V1-F	GTAACTTATGGGACTTGTACTACCAC
DV2-V1-Probe-FAM	CACTGAGCGCTTACTTCTACGCCC
DV2-V1-R	CACATGTGGAACGAGTGC
	
DV2-WT	
DV2-WT-F	TGGGTAACTTATGGGACTTGTA
DV2-WT-Probe-FAM	ACCACGGGAGAACATAGAAGAGAA
DV2-WT-R	CACATGTGGAACGAGTGC
	
DV2-total	
DV2-total-F	CACCATAATGGCAGCAATCC
DV2-total-Probe-Hex	ACGACACATTTCCAGAGAGCCCTG
DV2-total-R	ACAGCTGTCAGTAAGATGAAA

### Purification of DENV by sucrose gradient ultracentrifugation.

Five ridged bottles of Vero or VF-1 (70 to 80% confluent) were infected with DV4-WT or DV4-prM-E89N at an MOI of 0.01 in DMEM/F12 medium supplemented with 2% FBS for 3 days. Infection medium was replaced with DMEM/F12 serum-free medium, and the virus supernatant was harvested 2 and 4 days after the medium change. Supernatants were clarified by filtration through a 0.45-μm-pore-size filter. Filtrates were pooled and concentrated via tangential flow filtration using Pellicon2 Mini Cassette with Biomax 100-kDa membrane (Millipore) from 1 L to 45 mL. Concentrated supernatants were purified via eight-step (60, 55, 50, 45, 40, 35, 30, and 15%) sucrose gradient ultracentrifugation with 22 mL of supernatant in each tube spinning at 17,000 rpm for 18 h at 4°C. Fractions (1 mL) were collected and run on a TGX stain-free gel (Bio-Rad).

### Furin cleavage prediction.

Furin cleavage site efficiency was predicted using the PiTou software (29), providing amino acids from position P14-P6′ of the DENV furin cleavage sites.

### Statistical analysis.

Statistical analysis was carried out using GraphPad Prism version 9.0. The growth kinetics and maturation of DENV variants were compared to their corresponding wild type using two-way analysis of variance (ANOVA) with multiple comparisons. Neutralization titers of DENV variants were compared to their corresponding wild type using the Student *t* test. Significance symbols are indicated in the figures as follows: *, *P* < 0.05; **, *P*< 0.01; ***, *P* < 0.001; ****, *P* < 0.0001. The data are graphed as means ± standard deviations.
