# Supplementary figures and images for: Generation of Mature DENVs via Genetic Modification and Directed Evolution
Source: mBio. 2022 Apr 28;13(3):e00386-22. doi: 10.1128/mbio.00386-22 (PMC9239201; doi:10.1128/mbio.00386-22)

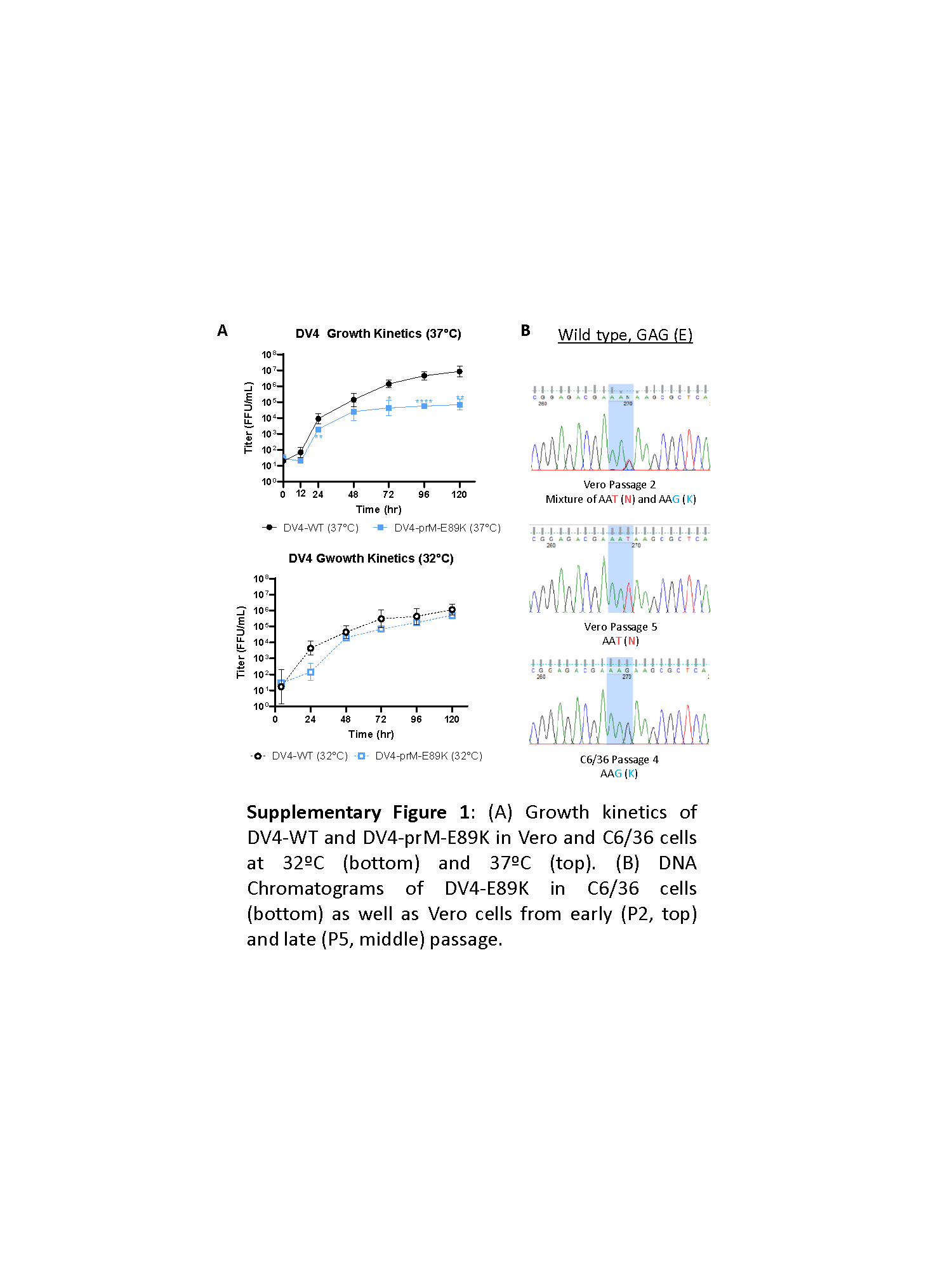

Supplement: FIG S1 [file mbio.00386-22-s0001.tif]

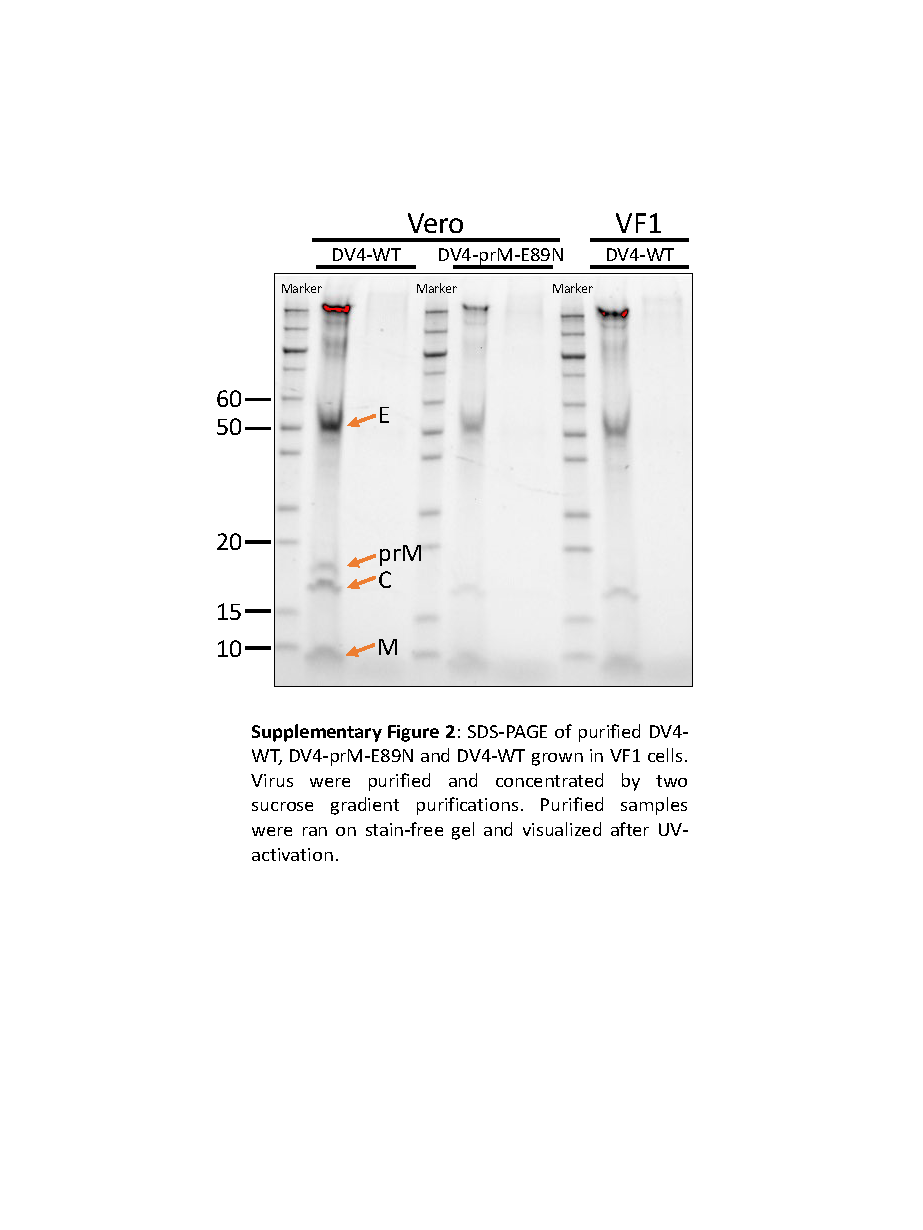

Supplement: FIG S2 [file mbio.00386-22-s0002.tif]

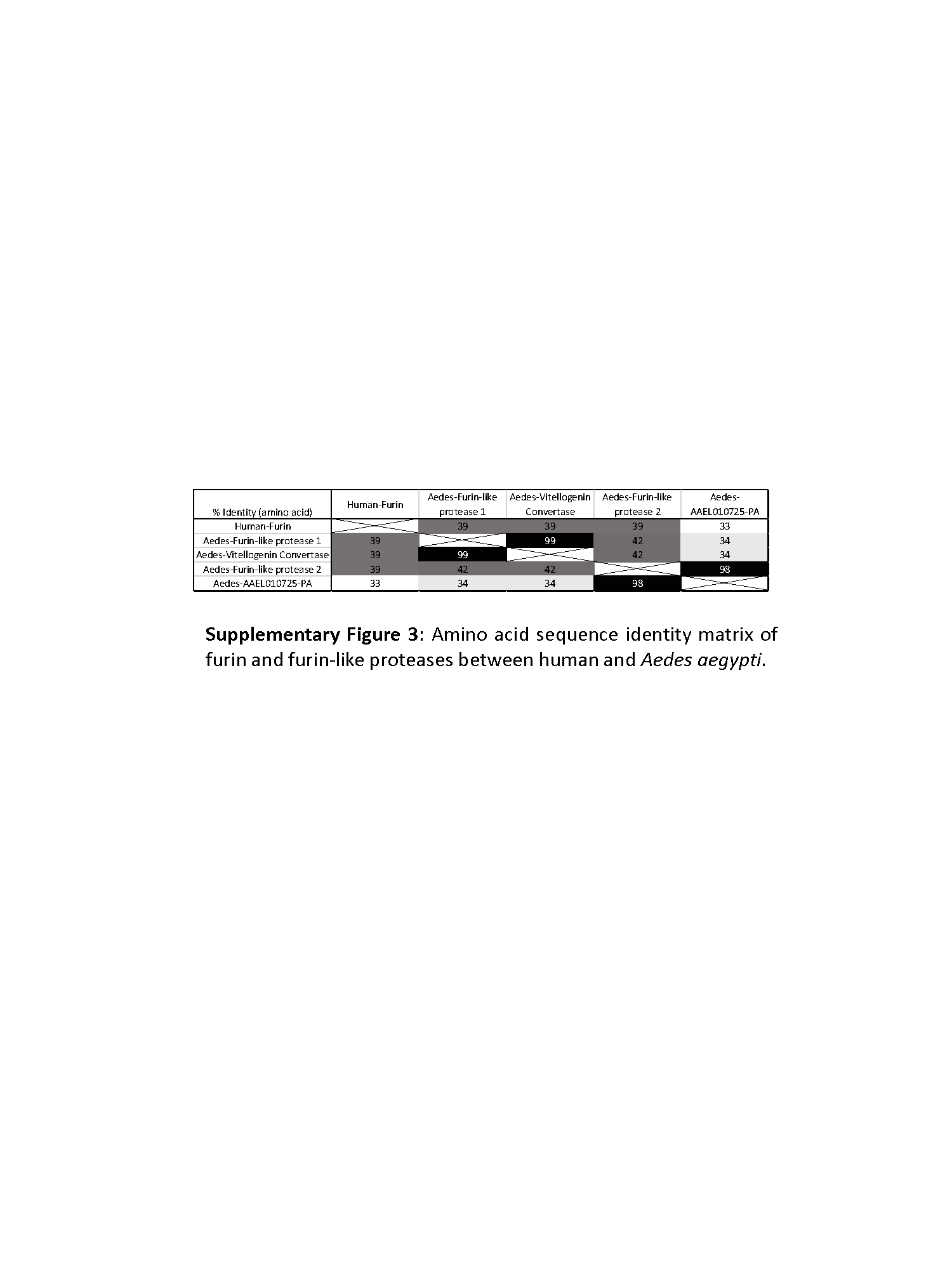

Supplement: FIG S3 [file mbio.00386-22-s0003.tif]
